# Prevalence of night work in the Brazilian employed population

**DOI:** 10.47626/1679-4435-2026-1621

**Published:** 2026-09-22

**Authors:** Claudio José dos Santos Júnior, Frida Marina Fischer

**Affiliations:** 1 Universidade de São Paulo, Faculdade de Saúde Pública, São Paulo, SP, Brazil

**Keywords:** night work, occupational epidemiology, workers’, health, National Survey of Health.

## Abstract

**Introduction:**

Night work and extended work schedules are among the leading occupational health risk factors.

**Objectives:**

To estimate the prevalence of night work among the employed Brazilian population.

**Methods:**

Cross-sectional, population-based study using data from the 2019 Brazilian National Survey of Health. Individuals aged 15 years or older who belonged to the economically active employed population were included. The outcome was self-reported night work, assessed using a survey question on work performed between 8:00 p.m. and 5:00 a.m. Responses were recorded as 0 = no and 1 = yes. Prevalence estimates and their 95% CIs were calculated accounting for the complex survey design according to sociodemographic, occupational, and state-level characteristics. Associations were evaluated using the Rao-Scott adjusted Pearson chi-square test.

**Results:**

The national prevalence of night work was 13.3% (95% CI, 12.8%-13.9%), ranging from 5.8% in Acre to 16.6% in Rio de Janeiro. Night work was more frequent in urban areas (14.2%), among men (16.3%), and among higher-income individuals (15.5%). The highest prevalences were observed in the accommodation and food services (28.7%) and transportation, warehousing, and postal services (26.9%) sectors.

**Conclusions:**

Approximately one in seven workers in Brazil engaged in night work. The prevalence varied according to regional, sociodemographic, and occupational characteristics, with higher prevalences in sectors requiring continuous operations. These findings underscore the importance of monitoring work schedules and implementing policies to protect the health of workers exposed to night work.

## INTRODUCTION

Night work and prolonged work schedules are among the major contemporary determinants of occupational health risk. Chronic exposure to shift work, including night shifts and irregular work schedules, is associated with circadian disruption [^1^], sleep disorders [^2^], increased susceptibility to accidents [^3^,^4^], cardiovascular [^5^] and metabolic [^6^] diseases, as well as adverse effects on mental health and quality of life.

The International Labour Organization and the World Health Organization recognize these forms of work organization as important occupational risk factors, with potential implications for productivity and social costs as well [^7^,^8^].

In Brazil, regulations governing working hours provide for additional compensation for night work, in addition to specific rights for employees engaged in shift work. The Brazilian Consolidation of Labor Laws defines urban night work as work performed between 10:00 p.m. and 5:00 a.m., with specific provisions for agricultural and livestock workers. For continuous rotating shift schedules, the law establishes a maximum workday of 6 hours, unless otherwise provided through collective bargaining. A minimum rest period must be observed between consecutive work shifts, in addition to a 24-hour paid weekly rest period, preferably including Sunday. Even under extended work schedules or special shift arrangements, employers must ensure compliance with statutory meal and rest breaks [^9^].

Despite this regulatory framework, informal employment practices [^10^,^11^], outsourcing [^12^], extended work schedules [^13^], multiple employment relationships, and atypical working hours remain widespread in Brazil, particularly in sectors that operate continuously, such as security services, public safety, communications, transportation, logistics, health, hospitality, and food services.

In middle-income countries such as Brazil, nationally representative studies on this topic remain scarce, and the magnitude and distribution of these forms of work organization are still poorly understood [^8^,^14^].

The available Brazilian literature is predominantly based on sector-specific, local studies or convenience samples, whereas broader nationally representative studies remain scarce. Most of the available evidence comes from studies conducted in specific occupational groups, such as health care, transportation, and public safety workers [^15^-^17^], or from cohorts in high-income countries. This lack of nationally representative data limits the development of work organization policies, occupational health surveillance, and regulatory planning.

This study aimed to estimate the prevalence of night work among the employed Brazilian population and to describe its distribution according to sociodemographic and occupational characteristics using data from a nationally representative health survey.

## METHODS

This was a cross-sectional, population-based study with national representativeness, conducted using microdata from the 2019 Brazilian National Survey of Health (Pesquisa Nacional de Saúde, PNS), developed by the Brazilian Institute of Geography and Statistics in partnership with the Ministry of Health.

The 2019 PNS was conducted throughout Brazil between August 2019 and March 2020. A complex three-stage cluster sampling design was used. In the first stage, census tracts (primary sampling units) were randomly selected; in the second stage, households were systematically selected within each census tract; and in the third stage, one household resident aged 15 years or older was randomly selected from each household to complete the individual questionnaire.

The study included individuals aged 15 years or older who belonged to the economically active employed population, defined as those engaged in paid work during the reference week of the interview. Observations with missing or undeclared data for the variables of interest were excluded.

The study outcome was night work, assessed using question M5c (M005010) of the PNS: “In your job(s), do you usually work at any time between 8:00 p.m. and 5:00 a.m.?” Responses were recorded as 0 = no and 1 = yes.

The independent variables included state of residence, area of residence (urban; rural), sex (male; female), age (in years: 15-29; 30-59; ≥ 60), race/skin color (White; Black; Asian; Brown; Indigenous), per capita household income (in Brazilian minimum monthly salaries: < 1; 1-3; > 3), employment category (domestic worker; military personnel - Army, Navy, Air Force, Military Police, and Fire Department; private-sector employee; public-sector employee; employer; self-employed worker; unpaid family worker), and primary economic activity (agriculture and extractive industries; manufacturing; construction; trade and motor vehicle repair; transportation, warehousing, and postal services; accommodation and food services; information, communication, financial, and administrative activities; public administration, defense, and social security; education, human health, and social services; other services; domestic services; and ill-defined activities).

Prevalence estimates and their corresponding 95%CI were calculated using the *svy* command in Stata/MP (Stata Corporation, College Station, USA), version 17.

Associations between the outcome and the independent variables were assessed using the Rao-Scott adjusted Pearson chi-square test for complex survey data.

All analyses incorporated the complex sampling design, which was specified using the *svyset* command in Stata/MP.

## Results

**Figure 1:**
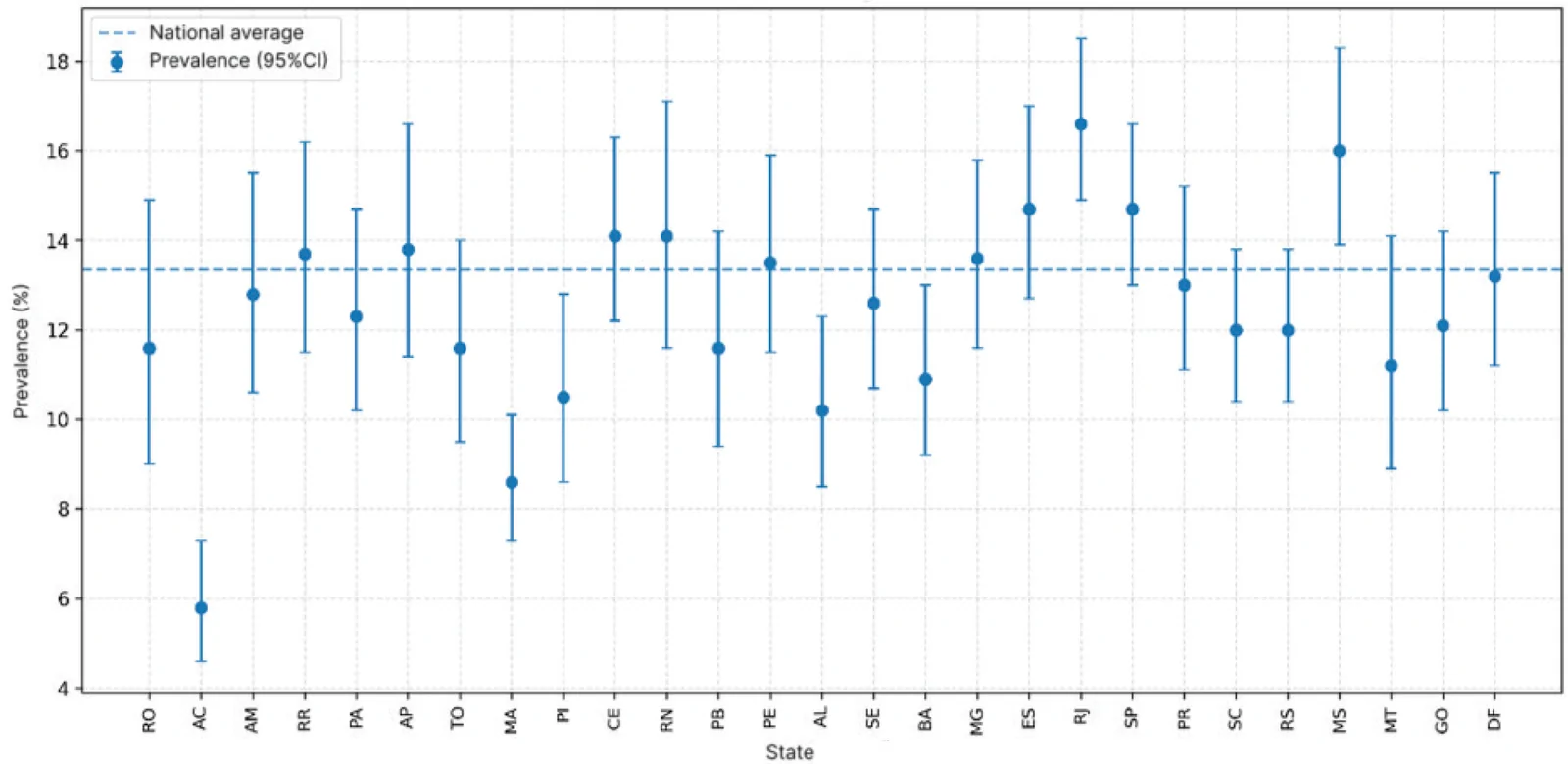
Prevalence of night work by state. Brazil, 2019-2020.

The prevalence of night work in Brazil was 13.3% (95%CI, 12.8%-13.9%), ranging from 5.8% in Acre to 16.6% in Rio de Janeiro (p < 0.001) (Figure 1).

AC = Acre; AL = Alagoas. AM = Amazonas; AP =Amapá; BA = Bahia; CE = Ceará; DF = Federal District; ES = Espírito Santo; GO = Goiás; MA = Maranhão; MG = Minas Gerais; MS = Mato Grosso do Sul; MT = Mato Grosso; PA = Pará; PB = Paraíba; PE = Pernambuco; PI = Piauí; PR = Paraná; RJ = Rio de Janeiro; RN = Rio Grande do Norte; RO = Rondônia; RS = Rio Grande do Sul; RR = Roraima; SE = Sergipe; SC = Santa Catarina; SP = São Paulo; TO = Tocantins.

In the demographic analysis, the prevalence of night work was higher in urban areas than in rural areas (14.2%; 95%CI, 13.6%-14.9% vs 7.3%, 95%CI: 6.5%-8.1%) (p < 0.001). Likewise, the prevalence was higher among men than among women (16.3%; 95%CI, 15.5%-17.2% vs 9.6%; 95%CI, 8.9%-10.4%) (p < 0.001). Prevalence also varied by age group, with the lowest prevalence observed among individuals aged 60 years or older (10.4%; 95%CI, 8.9%-12.0%) when compared with younger individuals (p = 0.004). In terms of income, participants with income greater than three Brazilian minimum monthly salaries had a higher prevalence (15.5%; 95%CI, 14.1%-16.9%) compared with those with lower incomes (11.6%; 95%CI, 10.9%-12.4%) (p < 0.001) (Table 1).

**Table 1:** Prevalence of night work according to sociodemographic and economic variables. Brazil, 2019-2020

Variable	Prevalence, % (95%CI)	p-value
Area of residence		< 0.001
Rural	7.3 (6.5-8.1)	
Urban	14.2 (13.6-14.9)	
Sex		< 0.001
Female	9.6 (8.9-10.4)	
Male	16.3 (15.5-17.2)	
Age (years)		0.004
15-29	13.8 (12.6-15.1)	
30-59	13.6 (12.9-14.2)	
≥ 60	10.4 (8.9-12.0)	
Race/skin color		0.317
White	13.3 (12.4-14.3)	
Black	14.5 (13.0-16.1)	
Asian	11.1 (7.6-16.1)	
Brown	13.1 (12.3-13.8)	
Indigenous	16.3 (10.6-24.1)	
Per capita household income (Brazilian minimum monthly salaries)		< 0.001
< 1	11.6 (10.9-12.4)	
1-3	14.5 (13.6-15.5)	
> 3	15.5 (14.1-16.9)	

According to primary economic activity, the prevalence of night work ranged from 5.2% (95%CI, 4.0%-6.8%) in construction to 28.7% (95%CI, 25.9%-31.6%) in accommodation and food services. High prevalences were also observed in transportation, warehousing, and postal services (26.9%; 95%CI, 24.1%-29.9%) and public administration, defense, and social security (19.5%; 95%CI, 17.4%-21.9%), whereas the lowest prevalences were found in agriculture and extractive industries (6.6%; 95%CI, 5.4%-7.9%) and domestic services (5.9%; 95%CI, 4.6%-7.5%) (p < 0.001) (Table 2).

**Table 2:** Prevalence of night work according to primary economic activity. Brazil, 2019-2020

Primary economic activity	Prevalence, % (95%CI)	p-value
Accommodation and food services	28.7 (25.9-31.6)	< 0.001
Transportation, warehousing, and postal services	26.9 (24.1-29.9)	
Public administration, defense, and social security	19.5 (17.4-21.9)	
Education, human health, and social services	15.7 (14.2-17.4)	
Information, communication, financial and administrative activities	15.5 (13.8-17.4)	
Manufacturing	14.8 (13.2-16.6)	
Other services	14.4 (12.1-16.9)	
Trade and motor vehicle repair	8.8 (7.8-9.9)	
Agriculture and extractive industries	6.6 (5.4-7.9)	
Domestic services	5.9 (4.6-7.5)	
Construction	5.2 (4.0-6.8)	

According to employment category, the prevalence of night work ranged from 12.0% (95%CI, 9.7%-14.7%) among employers to 14.1% (95%CI, 9.3%-20.8%) among military personnel, with no statistically significant differences across employment categories (p = 0.76) (Table 3).

**Table 3:** Prevalence of night work according to employment category. Brazil, 2019-2020

Employment category	Prevalence, % (95%CI)	p-value
Military personnel	14.1 (9.3-20.8)	0.76
Private-sector employee	13.7 (12.9-14.5)	
Public-sector employee	13.4 (12.0-14.8)	
Self-employed worker	13.3 (12.2-14.4)	
Unpaid family worker	12.6 (9.3-17.0)	
Domestic worker	12.3 (10.6-14.1)	
Employer	12.0 (9.7-14.7)	

## DISCUSSION

This nationally representative, population-based study estimated the prevalence and distribution of night work among the employed Brazilian population. Approximately one in seven workers (13.3%) engaged in night work. The distribution of this work schedule varied substantially across Brazilian states and according to sociodemographic characteristics and primary economic activity.

The overall prevalence of night work in Brazil was 13.3%. International studies likewise indicate that night work is widespread across countries. Nearly 19% of workers in the European Union perform night work at least once a month [^18^]. In the United States, 14.2% of the workforce engages in night work [^19^]. In China, 17.5% of workers perform night work at least once a month. In Japan, the average prevalence of night work has been reported to be 21.8% [^20^]. However, direct comparisons across countries should be interpreted with caution because legal definitions and methodological approaches used to characterize night work differ substantially [^20^].

The highest prevalence of night work was observed in Brazilian states characterized by high urbanization and strong participation in production and logistics networks, such as Rio de Janeiro, São Paulo, Espírito Santo, and Mato Grosso do Sul. This pattern is consistent with the economic structure of these states and the greater concentration of activities requiring continuous operations [^21^-^23^].

The analysis of demographic characteristics showed a higher prevalence of night work among urban residents, men, individuals in middle age groups, and workers with higher incomes.

The higher prevalence of night work among men may be partly explained by their greater concentration in occupations historically associated with night work, as well as by differences in labor market participation. A previous study supports this finding by showing that women are more likely to work daytime and evening shifts than men and, when engaged in night work, tend to prefer fixed shifts. According to the authors, this pattern may be explained, at least in part, by the multiple roles commonly assumed by women, including paid employment, household responsibilities, and caregiving for children or other dependents [^24^]. Furthermore, men are more frequently exposed to workdays exceeding 13 hours, rotating shift schedules, and prolonged workweeks [^24^] and experience a higher incidence of occupational injuries because of greater occupational exposure to workplace hazards [^4^,^25^].

The higher prevalence of night work in urban areas likely reflects the greater complexity of urban economies and the need to ensure the continuous provision of goods and services. Within this organizational context, production and service activities operate around the clock in cities and surrounding areas [^26^].

The observed income gradient suggests a higher prevalence of night work among individuals with higher incomes. This finding may be explained, at least in part, by financial compensation mechanisms, including night shift premiums and differential pay related to work schedules, which tend to increase workers’ earnings. Moreover, compensation structures associated with shift work, often linked to overtime pay, night shift differentials, and longer working hours [^9^], may contribute to the higher earnings observed among these workers.

The lower prevalence of night work among individuals aged 60 years or older may partly reflect reduced labor force participation in this age group, as well as lower exposure to night work resulting from the transition to retirement and age-related health limitations. International evidence supports this finding, indicating that older workers tend to reduce their exposure to night work because of its potential adverse effects on health and well-being [^24^].

Across primary economic activity groups, night work was particularly prevalent in the accommodation and food services and transportation and warehousing sectors, where occupational demands require around-the-clock activities. In contrast, construction, agriculture, and domestic services showed substantially lower prevalences, consistent with the predominance of daytime work schedules in these sectors.

International evidence likewise indicates that night work is more common in sectors requiring continuous operation, including the service, manufacturing, and health care sectors. In many of these settings, work organization requires around-the-clock operations and/or expanded activities during periods of lower urban circulation, which may contribute to the higher prevalence of night work [^27^].

From an occupational health perspective, the literature shows that night work is associated with circadian rhythm disruption, sleep restriction and fragmentation, hormonal and metabolic alterations, and increased risk of adverse physical and mental health outcomes [^20^]. Evidence also links night work to a higher occurrence of sleep disorders [^2^], depression and anxiety [^28^], dyslipidemia [^6^], and hypertension [^29^], as well as to a greater prevalence of unhealthy behaviors, such as lower levels of physical activity [^30^].

Although this study did not directly assess health outcomes, identifying sectors and occupational groups with a higher prevalence of night work may help inform occupational health surveillance strategies. Among the study’s main strengths are its national scope and population representativeness, ensured by the multistage probability sampling design of the PNS. Furthermore, the use of standardized survey data and sampling weights accounting for the complex survey design strengthens the robustness of the estimates and supports their generalizability to the employed Brazilian population.

Some limitations should be acknowledged. First, the cross-sectional design precludes causal inference, and the use of self-reported data may have resulted in exposure misclassification. Moreover, the definition of night work adopted by the PNS (8:00 p.m. to 5:00 a.m.) differs from that established in Brazilian labor legislation, which defines urban night work as work performed between 10:00 p.m. and 5:00 a.m. and specifies different time intervals for agricultural and livestock work (9:00 p.m. to 5:00 a.m. and 8:00 p.m. to 4:00 a.m., respectively). Consequently, the survey definition may have led to a slight overestimation of exposure prevalence, and this should be considered when interpreting the findings. Similarly, the absence of information on the duration and intensity of night work exposure precluded the assessment of potential dose-response relationships. Future longitudinal studies incorporating more detailed measures of work schedules are warranted to improve understanding of these aspects.

## CONCLUSIONS

Night work had an overall prevalence of approximately one in seven workers in Brazil. Its prevalence varied according to sociodemographic, regional, and primary economic activity characteristics, with higher prevalence observed in urban areas, among men, adults, and in sectors requiring continuous operations.

These nationally representative findings provide an empirical basis for monitoring work schedules and developing strategies to promote occupational health. Identifying the occupational groups and economic sectors with the highest prevalence of night work is essential for informing occupational health surveillance and guiding interventions aimed at reducing the risks associated with work schedule organization and improving working conditions in Brazil.

## Data Availability

The data are available in a repository (https://www.pns.icict.fiocruz.br/).
